# Improving the Diagnostic Potential of Extracellular miRNAs Coupled to Multiomics Data by Exploiting the Power of Artificial Intelligence

**DOI:** 10.3389/fmicb.2022.888414

**Published:** 2022-06-09

**Authors:** Alessandro Paolini, Antonella Baldassarre, Stefania Paola Bruno, Cristina Felli, Chantal Muzi, Sara Ahmadi Badi, Seyed Davar Siadat, Meysam Sarshar, Andrea Masotti

**Affiliations:** ^1^Research Laboratories, Bambino Gesù Children’s Hospital-IRCCS, Rome, Italy; ^2^Department of Science, University Roma Tre, Rome, Italy; ^3^Microbiology Research Center (MRC), Pasteur Institute of Iran, Tehran, Iran; ^4^Mycobacteriology and Pulmonary Research Department, Pasteur Institute of Iran, Tehran, Iran

**Keywords:** circulating miRNAs, fecal miRNAs biomarkers, urinary miRNAs detection, oral diagnostics, Machine Learning (ML), Artificial Intelligence (AI)

## Abstract

In recent years, the clinical use of extracellular miRNAs as potential biomarkers of disease has increasingly emerged as a new and powerful tool. Serum, urine, saliva and stool contain miRNAs that can exert regulatory effects not only in surrounding epithelial cells but can also modulate bacterial gene expression, thus acting as a “master regulator” of many biological processes. We think that in order to have a holistic picture of the health status of an individual, we have to consider comprehensively many “omics” data, such as miRNAs profiling form different parts of the body and their interactions with cells and bacteria. Moreover, Artificial Intelligence (AI) and Machine Learning (ML) algorithms coupled to other multiomics data (i.e., big data) could help researchers to classify better the patient’s molecular characteristics and drive clinicians to identify personalized therapeutic strategies. Here, we highlight how the integration of “multiomic” data (i.e., miRNAs profiling and microbiota signature) with other omics (i.e., metabolomics, exposomics) analyzed by AI algorithms could improve the diagnostic and prognostic potential of specific biomarkers of disease.

## Introduction

Over the past decade, the Human Microbiome Project has started many interconnected activities and projects, which allowed us to understand that we have “another” genome (i.e., the microbiome) (Human Microbiome Jumpstart Reference Strains Consortium et al., 2010; Qin et al., 2010). This topic boosted an incredible number of ever increasing interdisciplinary studies applied to medicine [Integrative HMP (iHMP) Research Network Consortium, 2019]. Therefore, the importance of gut microbiota is enormous, since it has been unraveled in these years that an imbalance of the microbial composition may lead to shift from the physiological state to dysbiosis, hence, from health to disease status (Althani et al., 2016). The microbial composition in the gut may be modulated also by a different diet (Del Chierico et al., 2014). In fact, different feeding modalities during the first months of life is among the factors that explain the high variability of the microbiota after birth (Putignani et al., 2014). Manipulation of intestinal microbes due to mode of delivery (cesarean section versus vaginal delivery), breast feeding/formula/mixed feeding, overuse/misuse of antibiotics, prebiotics and probiotics, dietary modification, regional lifestyle, and ultimately fecal microbiota transplantation are among the external factors influencing gut microbiota composition (Arrieta et al., 2014; Zhuang et al., 2019; Niu et al., 2020).

However, many other biologically relevant molecules (i.e., microRNAs or miRNAs) can modulate gut microbiota homeostasis and composition. In the last few years, the discovery of miRNAs in biological fluids has generated a great interest for their potential use as biomarkers. Moreover, many miRNAs are stably expressed in various body fluids and a recent paper reported a good correlation of circulating miRNAs in body fluids with that of tissue miRNAs, opening the way to use miRNAs as biomarkers to monitor corresponding specific human diseases (Cui and Cui, 2020).

Circulating biomarkers play a significant role in clinical applications especially for the diagnosis of specific diseases, to monitor the therapeutic effect of a drug or to predict the tumor recurrence in chemotherapy-treated patients (Kosaka et al., 2010). As an example, Chim and coworkers were the first to identify circulating placental miRNAs in the plasma of pregnant women (Chim et al., 2008). Circulating miRNAs have many of the essential characteristics of good biomarkers: they are stable in the circulation and resistant to digestion by RNAses, extreme pH, high temperatures, extended storage and multiple freeze-thaw cycles (Mitchell et al., 2008; Chen et al., 2009). In many cases, changes in circulating miRNA expression levels have been associated with different diseases or certain biological/pathological stages. Circulating miRNAs are released in the bloodstream into many forms, although their origin and the mechanism of their release have not been completely elucidated. The reasons of the high stability of circulating miRNAs remain largely unknown as well, although several hypothesis have been suggested (Cortez et al., 2011).

Despite the few information about the biogenesis and stability of circulating miRNAs, we know more about their functional role in health and disease, as briefly discussed in the following paragraphs.

## Serum/Plasma miRnas

Circulating miRNAs are small RNA molecules that can be detected, free or encapsulated in vesicles (i.e., exosomes), in many biological fluids (i.e., blood, serum, plasma, saliva, urine, etc.) (Weber et al., 2010). Since many years, circulating miRNAs are considered powerful diagnostic biomarkers for many diseases and the novel frontier of liquid biopsies (Pardini et al., 2019). It is quite impossible to cite all the papers dealing with circulating miRNAs in health and disease conditions, as many papers appeared in the literature but we will cite only a few of them related to pediatric diseases.

In neonatology, miRNAs from cord blood showed a very low correlation with maternal blood miRNAs expression but instead they appeared to be powerful early biomarkers of child health (Cretoiu et al., 2016; Dypas et al., 2020). Circulating miRNAs are also considered good diagnostic tools for example in celiac disease and its treatment (Felli et al., 2022) or to detect the evolution of liver diseases (Calkins et al., 2020; Resaz et al., 2021; Nagura et al., 2022).

Circulating miRNAs are also widely studied also in cancer disease (Colletti et al., 2019; Galardi et al., 2019). In cardiology, specific sets of miRNAs are able to distinguish patients with supraventricular (SVa) and ventricular (Va) arrhythmias from the controls (Moric-Janiszewska et al., 2021).

In neurological and neurodevelopmental child disorders, serum miRNAs have been used as biomarker for autism spectrum disorder (ASD) (Vasu et al., 2014), and temporal lobe epilepsy (TLE) in children (Niu et al., 2021).

Finally, the identification of specific circulating miRNAs in children with type-1 diabetes (T1D) allowed to discriminate early and late stages of diabetes (Erener et al., 2017; Margaritis et al., 2021), the glycemic status and the ongoing islet autoimmunity in high-risk individuals (Akerman et al., 2018). Circulating miRNAs can also predict metabolic diseases such as insulin resistance in obese pre-schoolers (Masotti et al., 2017).

Circulating miRNAs can act not only as “passive” biomarkers but to have also an “active” communication role in distant organs (Valadi et al., 2007). However, one of their most interesting role is to be part of a more complex network of communication mediated by exosomes. In particular, miRNAs have both a regulatory effect on pathogen infections (i.e., by mediating further infection by transmitting pathogen-related molecules, participating in the immune escape of pathogens and inhibiting immune responses by favoring immune cell apoptosis) and an anti-infection role (i.e., by inhibiting pathogen proliferation and infection directly or inducing immune responses such as those related to the function of monocyte-macrophages, NK cells, T cells, and B cells) (Zhang et al., 2018).

We have reported that HIV-1 Nef protein is secreted also within exosomes and contributes to regulate the intercellular communication exploiting the vesicular trafficking machinery of the host (Felli et al., 2017b). This can be considered as a potential inter-kingdom communication pathway between virus and humans, where viral Nef contributes to modulate and post-transcriptionally regulate the host gene expression and immune response. Therefore, we cannot exclude that these exosomes contain also miRNAs and that these circulating molecules might have a modulatory role, not exclusively in infections.

## Fecal miRnas

Recently, the novel concept of intestinal epithelial cells able to release luminal regulatory miRNAs (i.e., fecal miRNAs) was described (Masotti, 2012; Liu and Weiner, 2016; Felli et al., 2017a; O’Brien et al., 2018; Li et al., 2020; Sarshar et al., 2020b). Noticeably, less is known about the effect of bacterial pathogens on host miRNA expression, as well as the reciprocal effect (i.e., the role of host cell miRNAs on modulating bacterial infections). Interestingly, the expression of miRNAs have been recently reported to be correlated with the richness and diversity of the microbial community (Liu et al., 2016; Ragusa et al., 2020; Del Pozo-Acebo et al., 2021). Thus, miRNAs may enter bacteria and, in turn, affect their biological processes by regulating bacterial gene expression and growth, therefore, giving to pathogenic bacteria the opportunity to expand, leading to dysbiosis (Liu and Weiner, 2016; Liu et al., 2016; Wu et al., 2016). Liu and colleagues showed that fecal samples contained small RNA species (size ranging between ∼20–100 nt) similar to extracellular/exosomal RNA. They further suggested that fecal miRNAs, mainly released by intestinal epithelial cells (IEC) and Hopx-positive cells, regulate bacterial gene transcripts and affect bacterial growth such as *Fusobacterium nucleatum* and *Escherichia coli* (*E. coli*) (Liu et al., 2016). In addition, they found that gut dysbiosis in deficient IEC-fecal miRNA could be restored through WT fecal miRNA transplantation. This group later proposed the term of “miRNA-microbiome axis” as a potential therapeutic approach.

A recent study reported that IECs release miRNAs packed in exosomes and these IEC-exosomal miRNAs in turn influence the composition of gut bacterial populations (Kumar et al., 2021). Specifically, IEC-exosomes could be taken up by gut bacteria and inhibit the expression of the *E. coli tnaA* gene and its indole production, an intracellular signal in microbial communities mainly in Gram negative bacteria.

## Salivary miRnas and Oral Microbiota

For many years until now, blood has been considered the biological source of choice for the diagnosis of many diseases and for clinical monitoring. So far, no alternatives were considered valid and reliable. The concept of using less invasive methods, such as saliva (i.e., oral diagnostics) has been increasingly adopted in the clinical field owing to the presence of specific molecules or microorganisms that can be used as valuable biomarkers. In fact, these components are not only altered in oral diseases but also correlated with damaged tissues or distal organs. Therefore, oral fluids have been studied in details for both diagnostic and potential therapeutic applications. Five major constituents can be considered for these purposes: proteins, mRNAs, miRNAs, metabolites and microbes that can be altered alone or in combination according to the analyzed disease.

Some authors focused on salivary miRNAs as diagnostic biomarkers of hand, foot and mouth disease (HFMD) in pediatric patients, and they found that miR-221 was consistently and significantly downregulated in the patients cohort (Min et al., 2018) supporting the method of using salivary miRNAs to diagnose this infection. Other authors identified the salivary miR-4668 as a novel potential biomarker of eosinophilic esophagitis (Bhardwaj et al., 2020).

Recent evidences have demonstrated that salivary molecules, as well as bacterial populations, can be dysregulated by several pathological conditions including neuro-psychiatric diseases such as the elusive Autistic Spectrum Disorder (ASD) (Ragusa et al., 2020). The authors performed a combined approach of miRNA expression profiling and 16S rRNA microbiome analysis on saliva from 53 ASD and 27 neurologically unaffected control children. The authors found that miRNAs and microbes were statistically associated to different neuropsychological scores related to anomalies in social interaction and communication and among the prevalent miRNA/bacteria associations, the most relevant was the negative correlation between salivary miR-141-3p expression and *Tannerella* spp. abundance. Furthermore, the authors suggest that a potential cross-talk between circulating miRNAs and resident bacteria could occur in saliva of ASD.

To add further complexity to these relationships, the recent evidences that circadian changes may be in relation to, or affect, the host microbiota (i.e., gut microbiota) (Thaiss et al., 2016; Dickson, 2017) solicited Hicks et al. (2018) to investigate the daily oscillations in salivary miRNAs and microbial RNAs. They explored relationships between circadian oscillations in human salivary miRNAs and microbial RNAs that may have distinct implications in human health and disease (Hicks et al., 2018). However, all of these studies suggest the need of a reliable method to study this complex relationship.

In the last few years, the Salivaomics Knowledge Base (SKB) consisting in data repositories, management systems and dedicated web resources has been established to support human salivary research in these fields (i.e., proteomics, transcriptomics, miRomics, metabolomics and microbiota analysis) (Shah, 2018).

## Urinary miRnas and Microbiota

Urine is the metabolic fluid excreted through the urinary system and contains not only bacteria, but also exosomes and miRNAs, among the others (Weber et al., 2010). Urinary microbiota refers to the microbial community present in urines, and generally contains a different and less abundant microbial population compared to other sites of the body (i.e., the gut). Nevertheless, dysbiosis in the urinary tract leads to urinary tract infections (UTIs) such as cystitis, although the interaction between different microbiota ecosystems has been poorly investigated (Ceprnja et al., 2021; Martinez et al., 2021; Perez-Carrasco et al., 2021). However, we know that different species of *Lactobacillus* (i.e., *L. crispatus*, *L. gasseri*, *L. iners*, and *L. jensenii*) are dominant in the urinary tract and protect the host, and further maintain a good microbial balance. In women, a part of this microbiota is represented by *Gardnerella*, *Sneathia*, *Staphylococcus*, and *Enterobacteriacae* members (Brubaker and Wolfe, 2017), whereas in men, the urobioma includes mainly *Firmicutes*, *Actinobacteria*, *Proteobacteria* and *Bacteroidetes* (Nelson et al., 2010). Similarly to what already observed for gut microbiota, the urinary microbiota can be affected by external environmental factors that may alter its composition leading to dysbiosis. Interestingly, the discovery of exosomes present also in urine, referred as urinary exosomes, outlined some interesting properties of these vesicles.

UTIs, which include cystitis and pyelonephritis, are mainly caused by uropathogenic *E. coli* (UPEC) strains that originate from the intestinal microbiota through the migration to the perianal region and to the urinary tract (the fecal-perineal-urethral route) (Scribano et al., 2021) and the establishment of adaptive strategies during host cell adhesion (Sarshar et al., 2022). Several strategies (i.e., anti-adhesive molecules) have been suggested as good antibiotic alternatives to prevent UTIs (Sarshar et al., 2020a; Scribano et al., 2020). Interestingly, also urinary exosomes have the ability to inhibit the growth of, and kill, bacteria such as UPEC in the urinary tract (Hiemstra et al., 2014). Exosomes can also have an inhibitory role in viral infections while they may also facilitate the spread of viral (Caobi et al., 2020) or parasitic (Wu et al., 2019) infections. Since exosomes contain miRNAs, potent gene expression modulators, it is not surprising to observe these effects and expect to find circulating miRNAs in urine. In fact, Zhao and collaborators showed that there were a large number of differentially expressed miRNAs in urinary exosomes of type 2 diabetes mellitus (T2DM) as well as Diabetic kidney disease (DKD) patients, and that some of these urinary miRNAs are also able to predict early microalbuminuria (Zhao et al., 2020). A study reported by Scian and coworkers showed that urinary miRNAs are correlated with renal dysfunction and to pathophysiological processes. In fact, the authors investigated the expression profiles of miRNAs in patients with chronic renal transplant dysfunction both in kidney biopsies and in paired urine samples (Scian et al., 2011) and detected a differential expression for several miRNAs in renal biopsies, confirmed by the same dysregulated miRNAs in urines.

Another study by Yang and collaborators evaluated the expression changes of urinary miRNAs in the progression of diabetic nephropathy (DN) and observed a distinct profile of dysregulated miRNAs in these patients. This findings further suggested the potentiality of urine-specific miRNAs as novel biomarkers for the diagnosis of early stages of diabetic nephropathy (Yang et al., 2013).

In the oncology field, other studies validated the reliability of urinary miRNAs as biomarkers of diseases, such as in the case of esophageal cancers, where the urinary levels of five miRNAs were reported significantly higher in the adenocarcinoma and esophageal squamous cell carcinoma patient group compared to the control group (Okuda et al., 2021). In pancreatic adenocarcinomas, three urinary miRNAs were significantly overexpressed in patients with stage-I cancer compared to healthy individuals (Debernardi et al., 2015), whereas nine urinary miRNAs were able to distinguish early-stages of renal cell carcinomas (RCC) or progressive and non-progressive tumors (Di Meo et al., 2020).

## Artificial Intelligence and Systems Biology: From Diagnosis to Personalized Medicine

**FIGURE 1:**
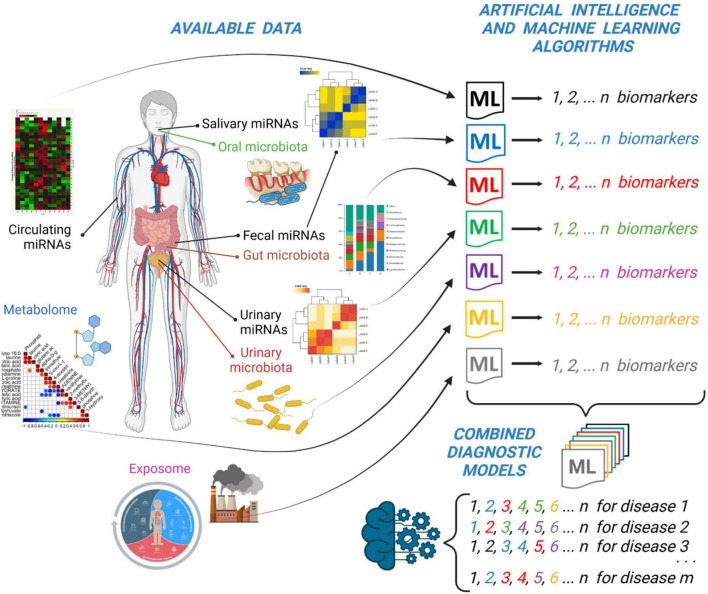
Integration of “multiomic” data (i.e., miRNAs profiling and microbiota analysis) together with metabolomic and exposomic analysis by Artificial Intelligence (i.e., ML) algorithms can lead to the discovery of sets of specific biomarkers of disease. A general model obtained by the integration of single sets of biomarkers could improve the diagnostic and prognostic ability of specific biomarkers of disease. This picture was created with BioRender.com.

In the previous paragraphs, we have discussed the recent findings reported in the literature about the use of miRNAs present in different biological fluids and specimens (i.e., serum, urine, saliva, stool) for diagnostic purposes. However, to have a more complete picture of the disease and to unravel novel biological and pathological links among different interactors, we think that we should analyze data coming from multiple “omics” techniques (i.e., circulating miRNAs, microbiome, metabolome, exposome, etc.) with a more integrated approach that takes into consideration the big-data produced by all of these techniques. The approach that we think feasible is to employ Artificial Intelligence (AI) and Machine Learning (ML) algorithms (Figure 1).

ML is defined as an ensemble of AI algorithms that enable a robust interrogation of multiple datasets aimed at identifying previously undiscovered patterns and relationships in the data. This approach has been applied successfully to the comprehension of neurodegenerative diseases and to the early diagnosis, prognosis and discovery of potential therapies (Myszczynska et al., 2020) or to predict the associations between miRNAs and diseases (Chen et al., 2017, 2018; He et al., 2018; Yao et al., 2019). Therefore, many different ML algorithms are available and depending on the data to analyze, it is essential to choose the most suitable one. In general, all of the approaches involve the division of the overall sample size in two subgroups: discovery and validation sets. ML algorithms have the potential to acquire information about the prognosis or to facilitate patient stratification and classification. The authors conclude by saying that large sets of curated datasets are needed to run ML algorithms and that one should rely on robust methods to obtain reliable diagnostic and prognostic models.

According to Lin and Lane, there is a huge demand for establishing algorithms in machine learning and systems genomics (MLSG) to establish genotype-phenotype relationships and find useful protocols for the discovery of diagnostics and therapeutics (Lin and Lane, 2017). Without going into details of the various algorithms employed in ML methods (i.e., naive Bayes, random forest, C4.5 decision tree, artificial neural networks, support vector machine, k-Means, k-nearest neighbors, and regression), we would like to present our personal view on the ideal approach that could be followed with miRNAs profiling and microbiota data by discussing some recent applications.

In serum/plasma miRNAs discovery, four distinct ML methods (i.e., the random forest wrapper method “Boruta,” the regression partition trees “Rpart,” LASSO regression, and the extreme gradient boosting “XGBoost”) and one univariate analysis have been employed to identify two (out of 20) circulating miRNAs (miR-636 and miR-187-5p) that have been found at the same time by all of the methods and that were able to diagnose with high accuracy patients with pulmonary arterial hypertension (PAH) (Errington et al., 2021). Moreover, the target genes of these two miRNAs were validated in two independent sets of human pulmonary artery smooth muscle cells, affording two genes differentially expressed *in vitro*, giving also some insights into the pathogenesis of PAH.

For urinary miRNAs, the majority of papers are related to cancer biomarker discovery (i.e., prostate cancers) and to the identification of small RNAs or miRNAs able to discriminate, for example, prostate cancers from benign prostate hyperplasia (BPH). In fact, Markert and collaborators used the random forest method to identify candidate small RNAs (Markert et al., 2021), and in other studies the same group focused on miRNAs (Holdmann et al., 2022).

Salivary miRNAs obtained by small RNA sequencing have been validated using ML with random forests as accessible and affordable biomarkers of alcohol dependence (AD) in a large group of 56 African-Americans and 64 European-Americans (Rosato et al., 2019). In another study, by applying support vector machine (SVM) models and leave-one-out cross-validation, the authors reported that salivary miRNAs were effective non-invasive biomarkers of hepatocellular carcinoma (Mariam et al., 2022).

Surprisingly, no studies dealing with fecal miRNAs and disease biomarker discovery have applied ML methods, but this can be easily explained by considering that fecal miRNAs represent a novel and emerging topic in the clinical diagnostic field.

On the contrary, both stool and salivary microbiota have been analyzed by ML algorithms. In a recent study, Saboo and collaborators aimed at determining the ability of stool vs. salivary microbiota to differentiate between cirrhosis and hepatic encephalopathy groups based on disease severity using ML. In particular, they classified stool and salivary microbiota using four different ML techniques (i.e., random forest, support vector machine, logistic regression, and gradient boosting) leading to the conclusion that stool microbiota are superior to saliva in distinguishing these two groups (Saboo et al., 2022).

Interestingly, in order to evaluate whether SARS-CoV-2 infected individuals should undergo hospitalization and reach the emergency department according to their symptoms, salivary metabolome have been correlated to serum biomarkers and salivary microbiota and analyzed by ML algorithms (Pozzi et al., 2022). Saliva and blood samples were collected over the course of two observational cohort studies. The results outlined that nine salivary metabolites are enough to assess the need of hospitalization. However, when these metabolites were combined with serum biomarkers and correlated with modulated microbiota taxa, just two salivary metabolites and one serum protein were sufficient to identify the patient to hospitalize. Therefore, this is a clear example that a combined omic analysis coupled to ML algorithms is advantageous to stratify patients affected by several diseases.

Therefore, we suggest to consider an AI method that could integrate the results of many ML algorithms applied to different profiles of miRNAs, microbiota or metabolites under investigation (Figure 1). This AI model should be ideally made by a combination of these single models obtained by single experiments. In this way, the distinctive circulating miRNAs obtained by one ML method can be listed together with fecal or urinary miRNAs or to other metabolites obtained by different ML methods. The overall “matrix” of data will be made by the *ensemble* of all of the potential biomarkers that will constitute a more robust model to predict a disease. It could be possible in the future to diagnose a disease (i.e., celiac disease) by simply looking at a couple of circulating miRNAs and a selection of specific fecal or urinary biomarkers (Paolini et al., 2022) or to the level of some metabolites obtained by analytical laboratory techniques (Figure 1).

## Other Potential Interactors to Complete the Overall Picture

**FIGURE 2:**
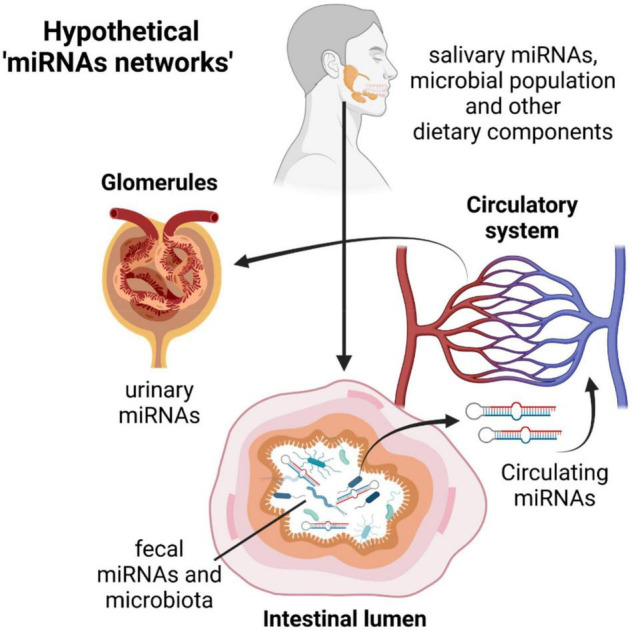
The miRNAs journey through the human body: from the mouth to the intestine through the digestive tract, migration from the gut to the circulatory systems and excretion by urine. The possible miRNA-host-pathogen cross talks and network of interactions during this journey could better classify the patient’s molecular characteristics (and the healthy/disease status) and drive clinicians to identify personalized therapeutic treatments. This picture was created with BioRender.com.

As reported by Liu, miRNAs and bacteria are functionally linked and a mutual host-guest regulation mediated by miRNAs and bacterial extracellular vesicles cannot be excluded. We also know that miRNAs produced in one tissue or circulating in serum/plasma is a direct consequence of what happens in distant organs, or they are a representation of a healthy/disease status. Therefore, we cannot exclude that circulating miRNAs, salivary miRNAs, urinary or fecal miRNAs can have a common denominator in describing the healthy/disease status of an individual and that a direct or indirect correlation may exist in different “districts” analyzed (Figure 2). Moreover, owing to the direct effects of miRNAs on bacterial gene regulation and therefore their function, as demonstrated by Liu, we can have multiple “regulatory pathways” coming from different parts of the human body that can have an overall beneficial or detrimental effect for human health (Figure 2).

Finally, it has been recently reported that also prokaryote cells are able to release outer membrane vesicles (OMVs) or extracellular vesicles (EVs) into the extracellular environments (Ahmadi Badi et al., 2020) and that specific isolation protocols can be used to characterize their structure (Sarra et al., 2020) and their small RNA content (Ahmadi Badi et al., 2020). This adds further complexity to the overall picture and another layer of possible interactions that should be considered by ML algorithms and investigated further.

For these reasons, AI approaches are needed not only to describe better the overall picture of a throughout screening of the patient, but also to unravel possible interactions among different districts, that are characteristics of the beginning or the progression of a certain disease. Moreover, AI and ML approach can help clinicians to classify the patient in a holistic way, by taking different analytical data and analyzing them in a coordinated and comprehensive way, and to define the best “category” where the patient belong to and opening the way to a laboratory-based personalized medicine approach. Finally, we foresee that the proposed strategy can be applied not only in the diagnostic field but also for the therapy of different adult and pediatric diseases and the follow-up of patients through the accurate identification and validation of the best miRNA targets (Major et al., 2020).

## Data Availability Statement

The original contributions presented in the study are included in the article/supplementary material, further inquiries can be directed to the corresponding author.

## Author Contributions

AP, AB, SPB, and CF drafted the manuscript. CM prepared the pictures. SAB and SS revised the manuscript. MS and AM drafted the manuscript and edited the final version of the manuscript. All authors contributed to the article and approved the submitted version.

## Conflict of Interest

The authors declare that the research was conducted in the absence of any commercial or financial relationships that could be construed as a potential conflict of interest.

## Publisher’s Note

All claims expressed in this article are solely those of the authors and do not necessarily represent those of their affiliated organizations, or those of the publisher, the editors and the reviewers. Any product that may be evaluated in this article, or claim that may be made by its manufacturer, is not guaranteed or endorsed by the publisher.
